# The effect of low-dose aspirin on the pregnancy rate in frozen-thawed embryo transfer cycles: A randomized clinical trial

**DOI:** 10.18502/ijrm.v13i9.7664

**Published:** 2020-09-20

**Authors:** Robab Davar, Soheila Pourmasumi, Banafsheh Mohammadi, Maryam Mortazavi Lahijani

**Affiliations:** ^1^Research and Clinical Center for Infertility, Yazd Reproductive Sciences Institute, Shahid Sadoughi University of Medical Sciences, Yazd, Iran.; ^2^Non-Communicable Diseases Research Center, Rafsanjan University of Medical Sciences, Rafsanjan, Iran.; ^3^Clinical Research Development Unit (CRDU), Moradi Hospital, Rafsanjan University of Medical Sciences, Rafsanjan, Iran.; ^4^Department of Obstetrics and Gynecology, Rafsanjan University of Medical Sciences, Rafsanjan, Iran.

**Keywords:** Aspirin, Embryo transfer, Pregnancy rates.

## Abstract

**Background:**

The results of previous studies on the effect of low-dose aspirin in frozen-thawed embryo transfer (FET) cycles are limited and controversial.

**Objective:**

To evaluate the effect of low-dose aspirin on the clinical pregnancy in the FET cycles.

**Materials and Methods:**

This study was performed as a randomized clinical trial from May 2018 to February 2019; 128 women who were candidates for the FET were randomly assigned to two groups receiving either 80 mg oral aspirin (n = 64) or no treatment. The primary outcome was clinical pregnancy rate and secondary outcome measures were the implantation rate, miscarriage rate, and endometrial thickness.

**Results:**

The endometrial thickness was lower in patients who received aspirin in comparison to the control group. There were statistically significant differences between the two groups (p = 0.018). Chemical and clinical pregnancy rates and abortion rate was similar in the two groups and there was no statistically significant difference.

**Conclusion:**

The administration of aspirin in FET cycles had no positive effect on the implantation and the chemical and clinical pregnancy rates, which is in accordance with current Cochrane review that does not recommend aspirin administration as a routine in assisted reproductive technology cycles.

## 1. Introduction

Human reproduction depends on a successful implantation and the development of an embryo on the endometrial surface. A receptive endometrium is a main factor for embryo implantation. Adjuvant therapy has been utilized to improve the thickness and vascularity of the endometrium with the purpose of improving implantation rates. One of this adjuvant therapies is low-dose aspirin administration (1).

There have been several studies on the effect of low-dose aspirin in assisted reproductive technology (ART) cycles, but contradictory and limited results have been reported on the effect of aspirin on the endometrial thickness as well as on the frozen-thaw embryo transfer (FET) cycles (2, 3).

Several studies showed that there was a direct correlation between an increased uterine vascular resistance and decreased endometrial and sub-endometrial blood flow with poor implantation and pregnancy rate (4-6). Aspirin can inhibit platelet aggregation and increases blood flow by changing the balance between thromboxane (which is a vasoconstrictor mediator) and prostaglandin (which is vasodilator mediator) (1). As per the recent studies, low-dose aspirin appears to be safe and well-tolerated by pregnant women (7, 8). Other aspirin mechanism is increasing it in the level of integrin B3 and Leukemia inhibitory factor (LIF) expression in the endometrium (9).

In a meta-analysis Wang and colleagues suggested that “low-dose aspirin may increase the pregnancy rate in In vitro fertilization/intra cytoplasmic sperm injection (IVF/ICSI) and recommended 100 mg/day ASA clinical use. Because of the limitation of the included studies, more well-designed large-scaled RCTs (randomized clinical trial) are necessary to confirm the effect of aspirin on the endometrial thickness in the FET cycles" (10).

Madani and colleagues performed a pilot clinical trial, where 60 patients who were candidates for FET cycle were divided into two groups. Group A received 100 mg aspirin orally and the other group received placebo. Finally, they reported that the administration of aspirin in FET cycles improved implantation rate, clinical pregnancy rate, and live birth rate. They also recommended that a large sample size is also necessary to confirm the effect of aspirin on the FET cycles (11). Fatemi and colleagues, on the other hand in a review study reported contradictory results about the effect of aspirin on the IVF cycles (12). Haapsamo and colleagues, reported that the administration of aspirin in ovarian stimulation cycles improved uterine hemodynamic status compared to the control group (13). In another study, the authors showed that the administration of aspirin in patients undergoing IVF cycle reduced uterine artery pulsatility index (PI) in the early and mid-pregnancy and reduced the risk of preeclampsia and intrauterine growth restriction (IUGR) (14).

In a systematic review, researchers concluded that there is insufficient evidence for routine administration of aspirin in IVF cycles. On the other hand, they reported that the administration of aspirin has no positive significant effects on IVF outcome and needs further studies (15). Dirckx and co-worker in Belgium found no positive effect of aspirin in improving clinical pregnancy in IVF cycles and recommended that it should not be used routinely (3). Also, in a recent Cochrane review, the authors concluded that the use of empirical aspirin for general IVF population cannot be recommended for routine use (16).

Based on the contradictory results and the lack of sufficient studies on the effect of aspirin on the pregnancy rate in FET, in our study we aimed to investigate the effect of low-dose aspirin adjuvant therapy in women undergoing FET cycles.

## 2. Materials and Methods

This study was a randomized clinical trial performed in the Research and Clinical Center for Infertility, Yazd Reproductive Sciences Institute from January 2019 to February 2020.

The inclusion criteria were age <40 yr, at least two frozen-thawed embryos available for another transfer, no contraindications for aspirin administration, no uterine disorders, no endometriosis, no history of uterine surgery, and no history of recurrent abortion (two or more than two abortions).

128 eligible women who were assigned for FET participated in the study. They were allocated into two groups randomly and equally. Group A (n = 64) was prescribed 80 mg aspirin, and for group B (n = 64), no treatment was prescribed to the routine FET protocols (Table I).

First, all patients underwent transvaginal ultrasonography on the second day of their cycles for excluding functional ovarian cysts. For endometrial preparation, patients received 6 mg estradiol valerate (aburahan co.) per day from second day of their cycles, and if the endometrial thickness was 7 mm or less on the thirteenth day of their cycles, the dose was increased to 10 mg.

When the optimal endometrial thickness (>8 mm) was obtained, vaginal progesterone 400 mg BID was started and continued until the 12 week of pregnancy. For endometrial thickness measurement, transvaginal ultrasonography (fillips affinity 70) was performed from the 13${}^{\mathrm{th}}$ day of their cycles and every 4 days if the endometrial thickness of 8 mm was not obtained on the 13th day.

The two selected embryos were transferred at cleavage stage by Cook catheter. 5000 IU HCG (human chorionic gonadotropin) was administered on the first, third, and sixth days of embryo transfer.

In our study 20 days after the embryo transfer beta human chorionic gonadotropin (βHCG) was checked. The primary outcome was clinical pregnancy rate that determined as presence of an intrauterine gestational sac with fetal heart beat (28-42 days after the embryo transfer). We requested women to continue taking aspirin for until 12 weeks of gestation. Secondary outcomes were implantation and miscarriage rates. The implantation rate was determined as the total number of gestational sacs per total number of transferred embryos, and the miscarriage rate was determined as the number of miscarriage that took place until the 20${}^{\mathrm{th}}$ week of pregnancy.

**Table 1:** Basic patient characteristics and clinical parameters in two study groups


**Variables**		**ASA group (n = 63)**	**Control group (n = 62)**	**P-value**
Age (Yr)*		29.48 ± 4.78	29.65 ± 4.52	0.835
BMI (kg/m ${}^{2}$)*				
	**≤ 24.9**	20 (31.25)	16 (25.00)	0.106
	**25-29.9**	30 (46.88)	38 (59.37)	
	**≥ 30**	14 (21.87)	10 (15.62)	
Duration of infertility (Yr)*		7.09 ± 3.49	7.37 ± 3.70	0.659
Number of previous embryo transfer cycles **				
	**1**	39 (60.9)	35 (54.7)	0.369
	**2**	23 (35.9)	25 (39.1)	
	**3**	2 (3.1)	4 (6.2)	
Cycle cancellation rate **		1 (1.6%)	2 (3.1)	1.00
Endometrial thickness (mm)*		8.64 ± 1.60	9.29 ± 1.70	0.028*
*Data presented as Mean ± SD (Student *t* test) ** Data presented as n (%) (chi-square test)				

### Ethical consideration

The study has been approved by the ethical committee of the Yazd Reproductive Sciences Institute (IR.SSU.RSI.REC.1398.003). All participants gave informed consent being included in the study.

### Statistical analysis

Data were analyzed using the Statistical Package for the Social Sciences 15.0 software. The baseline characteristics of the two groups of patients were compared using the student *t* test. Differences in the pregnancy outcomes of the two groups were analyzed using the Chi-square test. P ≤ 0.05 was considered statistically significant.

## 3. Results

**Figure 1:**
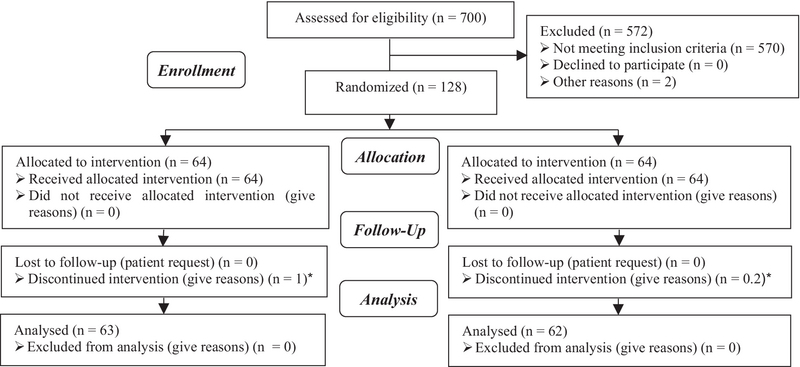
Consort flow chart.

In the present study, from 700 women who candidate for FET 572 were excluded from our study and we evaluated a total of 128 patients in two study groups: 64 cases in ASA (amino salicylic acid) group and 64 in control group. There was one cancelled cycle in case group (1/64) and two in control group (2/64) due to thin endometrium (< 7 mm) (Figure 1). As shown in table I, the age, BMI, infertility duration, and the number of previous embryo transfer cycles had similarity in the two study groups, and there was no statistically significant difference between the two groups. The endometrial thickness was lower in the group receiving ASA in comparison to the control group. The difference was statistically significant between the two groups (p = 0.028; Table I). ART outcomes in the two study groups are summarized in table II. The quality of transferred embryo was similar in the two groups and the highest number of embryos were in good quality (A and B). In all cycles, we transferred two embryo. From 126 transferred embryo in case group, 16 pregnancy sacs were observed in ultrasonography, and from 124 transferred embryo in control group, 18 pregnancy sacs were observed in ultrasonography. The Implantation rate was higher in the control group (14.5% vs. 12.6%), but this difference was not statistically significant. Chemical and clinical pregnancy rates were similar in the two groups, and there was no statistically significant difference.

**Table 2:** ART outcome in two study groups


**Variables**		**ASA group (n = 63)**	**Control group (n = 62)**	**P-value**
Embryo quality*				
	**A**	8 (12.7)	15 (24.2)	0.251
	**B**	38 (60.3)	32 (51.6)	
	**C**	17 (27.0)	15 (24.2)	
Implantation rate*		16/126 (12.69)	18/124 (14.51)	0.759
Chemical pregnancy rate*		19 (30.2)	15 (24.2)	0.547
Clinical pregnancy rate*		15 (23.8)	12 (19.4)	0.665
Abortion/clinical pregnancy**		0/15	0/12	
* Data presented as n (%). chi-square test ** data presented as n (%) Note: Data obtained by descriptive statistics; data presented number (%); P ≤ 0.05 considered statistically significant				

## 4. Discussion

The results of our study showed that there was no difference in the pregnancy outcome and the clinical pregnancy rate in patients who received aspirin in comparison with the control group in patients undergoing FET cycles. The endometrial thickness was significantly lower in the aspirin group than the control group. Some studies have investigated the role of aspirin in ART cycles and reported contradictory results (17, 18).

The majority of previous studies investigated the role of aspirin as an adjuvant therapy on ART outcome in fresh cycles (stimulated cycles) (17, 19). In fresh cycles, the estradiol levels are higher than in the freeze-thaw cycles. Also, in a fresh cycle, embryo is exposed to supra-physiological levels of estrogen during implantation but in the frozen-thaw cycles endometrial preparation is done artificially by estrogen and progesterone administration and the embryo is not exposed to supra-physiological level of estrogen. Supra-physiologic levels of estrogen may have a negative impact on endometrial receptivity and maybe responsible for implantation failure in ART (20, 21). Therefore, the results of aspirin adjuvant therapy in the fresh and frozen cycles are noncomparable. The results of the past studies on the effect of low-dose aspirin in FET cycles are limited.

The result of ART outcome in the present study was in contrast with Madani's study. They reported that the administration of aspirin in FET cycles improved the implantation, clinical pregnancy, and live birthrates, however, we found no statistically significant improvement in the biochemical, clinical pregnancy, and abortion rates in the aspirin group in comparison with the control. However, their study was a pilot study and the difference between the results of our study and the Madani's study may be the low sample size of their study (11).

Hsieh and colleagues showed higher clinical pregnancy rate and better endometrial pattern in patients with thin endometrium after aspirin administration, and because in this group they selected patients who had thin endometrium their results is in contrast to our findings (22).

Conforming to our study, a meta-analysis (15) and reports by Cochrane reviews (16, 23) also establish that there was no improvement in pregnancy rates with the use of low-dose aspirin. Jeromeh demonstrated that the clinical pregnancy rate was lower in the aspirin group compared with control group (11.1% vs 33.3% respectively) for, and the implantation rates were 2.9 and 10.9%, respectively. In this study, low-dose aspirin administration did not cause positive effect on pregnancy rates in FET cycles (24). Gelbaya and co-workers suggested that in FET cycles, there was no significant difference in pregnancy rate between untreated women with normal uterine perfusion and those that uterine perfusion was improved after aspirin administration. This result is similar with our study (25). In another study, Doppler ultrasound was used for uterine perfusion assessment and patients were classified as two groups: normal uterine perfusion and impaired uterine perfusion. administration of low-dose aspirin to hormone replacement therapy in women with impaired uterine perfusion was associated with improved uterine perfusion and acceptable pregnancy rates but without any benefits in patients with normal perfusion (26). In our study, the assessment of uterine perfusion and patients classification was not done.

## 5. Conclusion

Our study concludes that the administration of aspirin in frozen-thaw cycles had no positive effect on the implantation, chemical, and clinical pregnancy rates, and is in accordance with the current Cochrane review that does not recommend aspirin administration as a routine in ART cycles.

##  Conflict of Interest 

The authors have no financial or nonfinancial conflicts of interest.
